# Non-Histone Lysine Crotonylation Is Involved in the Regulation of White Fat Browning

**DOI:** 10.3390/ijms232112733

**Published:** 2022-10-22

**Authors:** Yuexia Liu, Yizhou Li, Juntong Liang, Zhuwen Sun, Chao Sun

**Affiliations:** College of Animal Science and Technology, Northwest A&F University, Xianyang 712100, China

**Keywords:** obesity, LC-MS/MS, non-histone, white fat browning, crotonylation modification

## Abstract

Lysine crotonylation modification is a novel acylation modification that is similar to acetylation modification. Studies have found that protein acetylation plays an important regulatory part in the occurrence and prevention of obesity and is involved in the regulation of glucose metabolism, tricarboxylic acid cycle, white fat browning and fatty acid metabolism. Therefore, we speculate that protein crotonylation may also play a more vital role in regulating the browning of white fat. To verify this conjecture, we identified 7254 crotonyl modification sites and 1629 modified proteins in iWAT of white fat browning model mice by affinity enrichment and liquid chromatography–mass spectrometry (LC-MS/MS). We selected five representative proteins in the metabolic process, namely glycerol-3-phosphate dehydrogenase 1 (GPD1), fatty acid binding protein 4 (FABP4), adenylate kinase 2 (AK2), triosephosphate isomerase 1 (TPI1) and NADH dehydrogenase (ubiquinone) 1 alpha subcomplex 8 (NDUFA8). Through qPCR, Western blotting, immunofluorescence staining, Oil Red O staining and HE staining, we demonstrated that GPD1 and FABP4 inhibited white fat browning, while AK2, TPI1 and NDUFA8 promoted white fat browning. GPD1 and FABP4 proteins were downregulated by crotonylation modification, while AK2, TPI1 and NDUFA8 proteins were upregulated by crotonylation modification. Further detection found that the crotonylation modification of GPD1, FABP4, AK2, TPI1 and NDUFA8 promoted white fat browning, which was consistent with the sequencing results. These results indicate that the protein crotonylation is involved in regulating white fat browning, which is of great significance for controlling obesity and treating obesity-related diseases.

## 1. Introduction

Protein post-translational modifications (PTMs) diversify protein structure, thereby increasing the diversity of protein functions. With the improvement in the sensitivity of mass spectrometry and the application of mutant animal models, the complexity of PTM regulation and its effect on protein function has been further studied. In 2011, a novel protein acylation modification was discovered, which was histone lysine crotonylation modification [1]. Crotonyl was found to be similar in structure to acetyl, with only one more carbon–carbon double bond than acetyl [2]. Further research found that the p300/CBP enzyme system promotes protein crotonylation, and histone crotonylation promotes gene transcriptional regulation [3,4]. At the same time, non-histone crotonylation modification exists in HeLa cells and regulates various protein functions and cellular processes [5]. Bioinformatic analysis revealed that crotonylated proteins were particularly enriched in nuclear proteins which are involved in RNA processing, nucleic acid metabolism, chromosome organization and gene expression [6].

White fat browning was considered as an approach to resist obesity [7]. Positive regulatory domain zinc finger region protein 16 (PRDM16) has be found to be an important transcription factor in inducing the differentiation of brown adipocytes, and it also activates the transcriptional coactivators proliferator-activated receptor-gamma coactivator-1-alpha (PGC-1α) and proliferator-activated receptor-gamma coactivator-1-beta (PGC-1β) of peroxisome proliferator-activated receptor γ (PPARγ), regulates mitochondrial function and promotes white adipocyte browning [8]. At the same time, studies have shown that PGC-1α can promote the expression of fibronectin type III domain-containing 5 (FNDC5) and increase irisin, thereby promoting the expression of uncoupling protein 1 (UCP1) in adipose tissue and the browning of white adipocytes [9]. Therefore, research on drugs targeting white fat browning or its regulators may be effective in the treatment of obesity, diabetes and related metabolic diseases.

Glycerol-3-phosphate dehydrogenase 1 (GPD1) is an NAD-dependent dehydrogenase that catalyzes the redox reaction between dihydroxyacetone phosphate (DHAP) and α-glycerol phosphate, which is reversible and oxidizes NADH to NAD^+^ [10]. In adipose tissue, GPD1 is an essential enzyme that provides the glycerol groups required for triglyceride synthesis [11]. There was a positive correlation between the enzymatic activity of GPD1 and body mass index (BMI) [12]. Meanwhile, the expression of GPD1 in skeletal muscle increased with obesity and decreased with weight loss caused by gastric bypass surgery [13]. It was found that short-term intake of a high-fat diet (HFD) increased the expression level of GPD1 in rat epididymal adipose tissue [14]. Moreover, hypoxia-inducible factor 1 subunit alpha (HIF-1α) directly regulates GPD1 at the transcriptional level, thereby inhibiting mitochondrial function and lipid metabolism [15]. In addition, GPD1 deficiency induces enhanced fat oxidation and inhibits weight gain [16].

Fatty acid-binding proteins (FABPs) are members of a family of highly conserved cytoplasmic proteins with high affinity for long-chain fatty acids and other hydrophobic ligands. FABPs are proteins that reversibly bind fatty acids and other lipids; nine tissue-specific cytoplasmic FABPs have been identified [17]. Fatty acid-binding protein 4 (FABP4), a member of the intracellular lipid-binding protein family, is an intracellular lipid chaperone that is mainly expressed in adipose tissue [18]. FABP4 is an adipokine that coordinates lipid transport in mature adipocytes [19], participates in crosstalk between adipose and peripheral tissues and promotes insulin resistance [20]. Meanwhile, FABP4 is a carrier protein of fatty acids and is involved in fatty acid uptake, transport and lipid metabolism [21], and variation mutations in FABP4 affect fat deposition in mammals [22].

Adenylate kinase 2 (AK2) is an isoenzyme of the AK family and plays an important role in energy metabolism control and mitochondrial function [23]. AK2 is a mitochondrial kinase that catalyzes the exchange of nucleotide phosphate groups, thereby participating in adenine nucleotide homeostasis [24]. It has been found that AK2 is significantly induced during adipocyte and B-cell differentiation. Moreover, AK2 induces the connection between mitochondrial energy metabolism and UPR response [25]. AK2 depletion results in mitochondrial dysfunction marked by early mitochondrial depolarization and reactive oxygen species production, while disrupting the oxidative phosphorylation pathway [26].

Triosephosphate isomerase 1 (Tpi1) is a centrally conserved glycolytic enzyme that catalyzes the interconversion of DHAP and glyceraldehyde 3-phosphate [27]. In humans, reduced TPI1 activity increases the specific antioxidant capacity of model organisms [28]. It has been found in hepatocellular carcinoma (HCC) cells that TPI1 inhibits growth, migration and invasion [29]. Daidzin interferes with the glycolysis/gluconeogenesis pathway by downregulating TPI1, thereby inhibiting the survival of HCC cells [30].

The full name of NDUFA8 is NADH dehydrogenase (ubiquinone) 1 alpha subcomplex subunit 8 (NDUFA8). Polymerase chain reaction analysis of rodent/human somatic cell hybrids mapped the human NDUFA8 gene to chromosome 9 [31]. Homozygous variants in NDUFA8 are associated with developmental delay, microcephaly and epilepsy due to deficiency of mitochondrial complex I. NDUFA8 deletion results in severe defects in mitochondrial complex I assembly, resulting in progressive neurological and developmental abnormalities [32]. Likewise, NDUFA8 deficiency causes altered mitochondrial morphology, loss of CI, impaired supercomplex formation and very mild disease progression [33].

## 2. Results

### 2.1. Construction and Validation of the White Fat Browning Mouse Model

**Figure 1:**
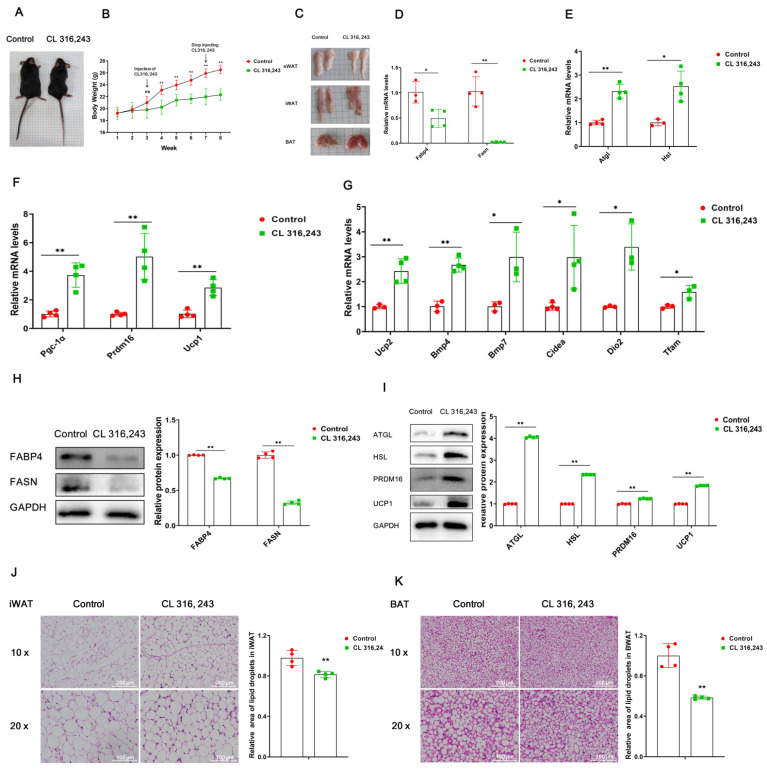
Construction and validation of the white fat browning mouse model. Note: The mice were injected with CL316,243 (1 mg/kg) every two days for 4 weeks, and after 2 weeks of stabilization, total RNA and total protein were extracted from iWAT of the mice. (**A**) Morphological diagram of mice (*n* = 5). (**B**) Change curve of mouse body weight (*n* = 5). (**C**) Morphological diagram of eWAT, iWAT and BAT (*n* = 5). (**D**–**G**) mRNA expression levels of genes related to lipid synthesis, lipolysis, white lipid browning and mitochondria (*n* = 4). (**H**,**I**) Protein expression levels of genes related to lipid synthesis, lipolysis and white lipid browning (*n* = 3). (**J**,**K**) HE staining of mouse iWAT and BAT, bar: 200 μm (*n* = 4). Values are mean ± SD compared to controls. * *p* < 0.05, ** *p* < 0.01.

We have constructed a white fat browning mouse model by intraperitoneal injection of β3-adrenergic receptor agonist (CL316,243, 1 mg/kg) into C57BL/6J mice, while raising vehicle control mice (Control) under the same conditions. We found that CL316,243 mice were significantly smaller in size than their controls (Figure 1A). Body weight analysis showed that the body weight of CL316,243 mice was significantly lower than that of their controls (Figure 1B). Of note, we have observed that the epididymal white adipose tissue (eWAT) and inguinal subcutaneous white adipose tissue (iWAT) of mice were reduced after injection of CL316,243, while the BAT (brown adipose tissue) was red-brown and increased in size (Figure 1C). Afterward, RNA and protein were extracted from iWAT. We found that compared with Control mice, the expression of genes Fabp4 and Fasn related to lipid synthesis was significantly decreased in CL316,243 mice (Figure 1D), and the expression levels of lipolysis-related genes Atgl and Hsl; white fat browning-related genes Pgc-1α, Prdm16 and Ucp1; and mitochondria-related genes Ucp2, Bmp4, Bmp7, Cidea, Dio2 and Tfam were significantly increased, which was detected by qPCR (Figure 1E–G). In addition, the detection results of Western blot and qPCR were consistent (Figure 1H,I), which preliminarily indicated that the white fat browning model mice were successfully constructed. Furthermore, HE staining of iWAT showed that lipid droplets in the adipose tissue of CL316,243 mice decreased significantly (Figure 1J). Meanwhile, the results of HE staining of brown adipose tissue also showed that lipid droplets were significantly reduced after injection of CL316,243 (Figure 1K). Based on the above results, the white fat browning mouse model was successfully established.

### 2.2. Sequencing of Non-Histone Crotonyl Modifications

**Figure 2:**
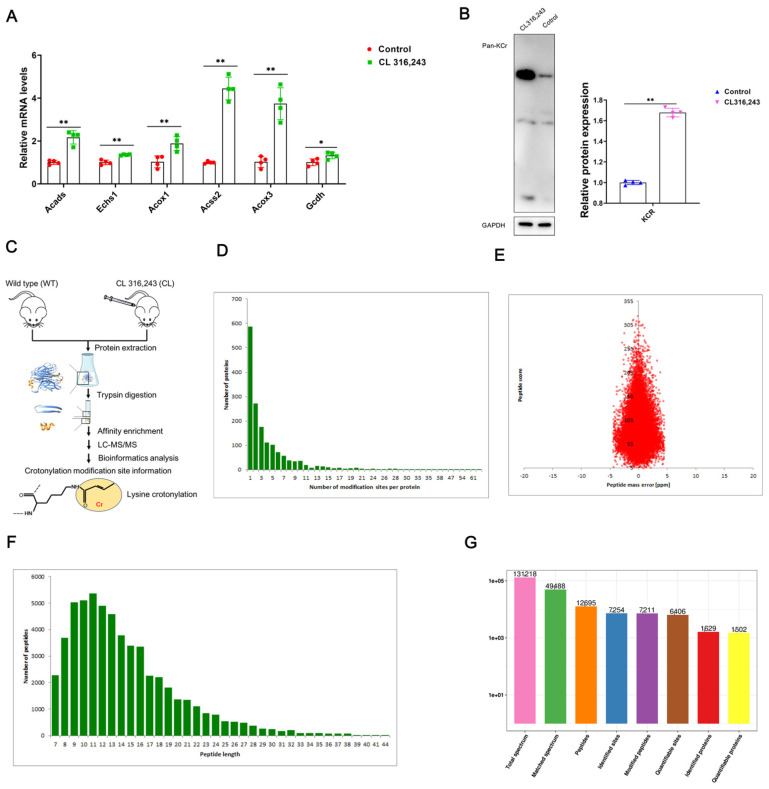
Sequencing of non-histone crotonylation modifications. Note: After the mice were injected with CL316,243 for 4 weeks and stabilized, the mouse iWAT samples were extracted and sent to the company for affinity analysis and LC-MS/MS, combined with database search and bioinformatics analysis. (**A**) The mRNA expression levels of crotonylation-related genes acyl-CoA dehydrogenase short chain (Acads), enoyl-CoA hydratase, short chain 1 (Echs1), acyl-CoA oxidase 1 (Acox1), acyl-CoA synthetase short chain family member 2 (Acss2), acyl-CoA oxidase 3 (Acox3) and glutaryl-CoA dehydrogenase (Gcdh) [34] (*n* = 4). (**B**) Protein crotonylation modification level (*n* = 4). (**C**). Schematic diagram of label-free quantitative proteomics of crotonylation. (**D**–**F**) Detection and analysis of sample quality (*n* = 3). (**G**) LC-MS/MS spectral library search analysis summary, overview of differentially modified modification sites (modified proteins), standardized differential modification site (modified protein) summary (*n* = 4). * *p* < 0.05, ** *p* < 0.01.

In order to further explore the intrinsic link between protein crotonylation modification and white fat browning, we injected mice with CL316,243 and found that the expression of crotonylation-related genes Acads, Echs1, Acox1, Acss2, Acox3 and Gcdh was significantly upregulated in CL316,243 mice (Figure 2A); meanwhile, the level of protein crotonylation was also significantly upregulated (Figure 2B), suggesting that there may be a close intrinsic relationship between white fat browning and protein crotonylation modification. In order to investigate the intrinsic relationship between white fat browning and protein crotonylation modification, we used affinity analysis and LC-MS/MS to assess the collected mouse iWAT, and we combined these analyses with database search and bioinformatics analysis to perform label-free quantitative proteomics of crotonylation modifications (Figure 2C). The detection revealed that compared with the control group, the number of proteins with less than five crotonylation modification sites accounted for the most after CL316,243 injection (Figure 2D). At the same time, the mass offset of all identified peptides has been detected, and we found that the peptide mass errors were centered at 0 and concentrated in the range of 10 ppm (Figure 2E). Moreover, the majority of the peptide lengths were distributed in the range of 8–20 amino acid residues (Figure 2F), which was in accordance with the rule of trypsin digestion of peptides, indicating that the sample preparation met the standard and could be used for subsequent experiments (Figure 2D–F). Furthermore, compared with the control group, 7211 peptides with crotonylation, 1629 proteins with crotonylation and 7254 crotonylation sites were identified after CL316,243 injection (Figure 2G). 

### 2.3. Non-Histone Crotonylation Modulates White Fat Browning

**Figure 3:**
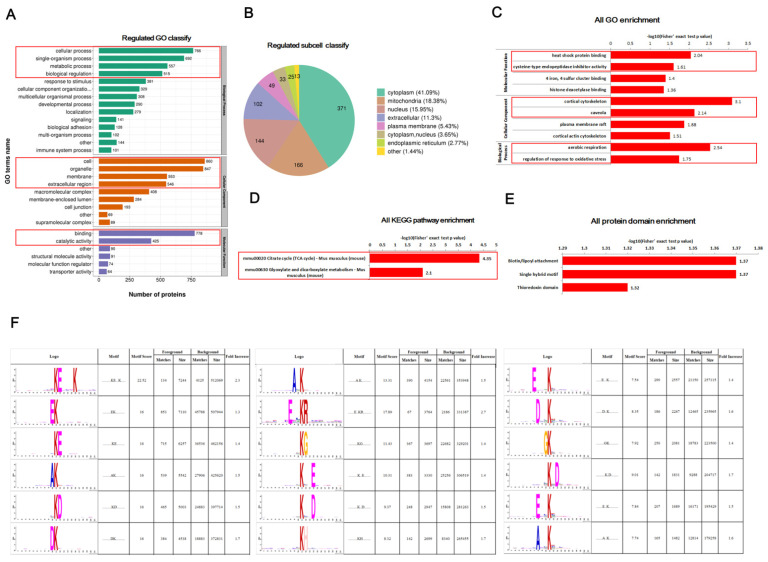
Non-histone crotonylation modification regulates white fat browning. Note: (**A**) GO secondary annotation of crotonylated proteins; (**B**) subcellular localization of crotonylated proteins; (**C**–**E**) enrichment analysis of crotonylated protein GO, KEGG and crotonylated modified domains; (**F**) crotonylation modification sequence motif of ±10 amino acids around the Kcr site.

GO analysis revealed that crotonylated proteins were mainly involved in biological processes such as cellular processes, single biological processes, metabolic processes and biological regulation; were mainly involved in the composition of cellular components such as cells, organelles, membranes, extracellular areas, macromolecular complexes, membrane-enclosed cavities and cell junctions; and mainly played molecular functions such as adhesion and catalytic activity (Figure 3A). The subcellular localization showed that crotonylated proteins were mainly located in the cytoplasm, mitochondria, nucleus, extracellular areas and other locations (Figure 3B).

Next, we performed an analysis of the enrichment of functions based on Gene Ontology (GO), Kyoto Encyclopedia of Genes and Genomes (KEGG) and protein domain database for the proteins screened by label-free quantitative proteomics. GO enrichment analysis showed that total crotonylated proteins were significantly enriched in molecular functions such as heat shock protein binding and cysteine-type endopeptidase inhibitor activity, in cellular components such as cortical cytoskeleton and pits, and in biological processes such as aerobic respiration and regulation of oxidative stress responses (Figure 3C). KEGG pathway enrichment analysis revealed that total crotonylated proteins were markedly enriched in the tricarboxylic acid cycle (TCA cycle), glyoxylate and dicarboxylate metabolism pathways (Figure 3D). Protein domain enrichment analysis indicated that total crotonylated proteins were mainly attached to and singly mixed with biotin acyl and were significantly enriched in thioredoxin domains (Figure 3E).

We used protein motif analysis to calculate the regularity trend of amino acid sequences in the region of crotonylation sites by counting the regularity of amino acid sequences before and after all crotonylation sites in the sample. The results of motif analysis showed that protein crotonylation modifications were most enriched at the amino acid sequences characteristic of ……KE……K…… (Figure 3F).

### 2.4. GPD1 and FABP4 Inhibit White Adipocyte Browning, While AK2, TPI1 and NDUFA8 Promote White Adipocyte Browning

**Figure 4:**
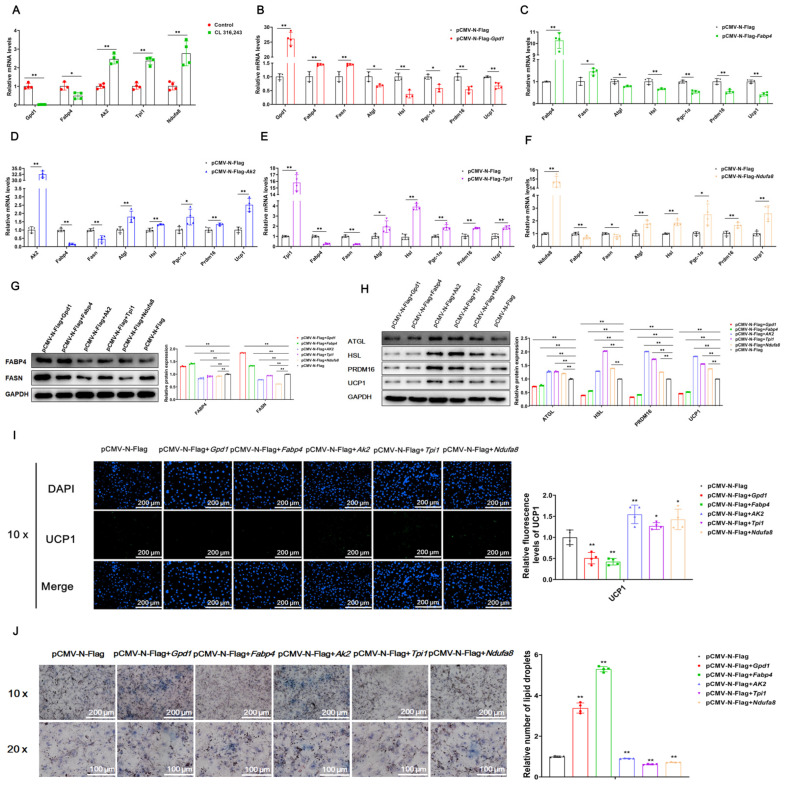
Gpd1 and Fabp4 inhibit white adipocyte browning, while Ak2, Tpi1 and Ndufa8 promote white adipocyte browning. Note: 3T3-L1 adipose precursor cells were induced to differentiate into mature adipocytes, and the constructed overexpression vector was transferred into adipocytes. Cellular RNA and protein were extracted after 24 h and 48 h, respectively. The expression efficiency of genes; the relative expression levels of lipid synthesis-related genes Fabp4 and Fasn; the relative expression levels of lipolysis genes Atgl and Hsl; and the relative expression levels of white fat browning-related genes Pgc-1α, Prdm16 and Ucp1 were detected. (**A**) After CL316,243 treatment of mice, the relative expression levels of each target gene mRNA (*n* = 4). (**B**–**F**) The relative mRNA expression levels of genes related to expression efficiency, lipid synthesis, lipolysis and white fat browning after overexpression of Gpd1, Fabp4, Ak2, Tpi1 and Ndufa8 (*n* = 4). (**G**,**H**) Relative protein expression levels of lipid synthesis-, lipolysis- and sebaceous browning-related proteins after overexpression of Gpd1, Fabp4, Ak2, Tpi1 and Ndufa8 (*n* = 3). (**I**) Immunofluorescence staining and quantification of Ucp1 after overexpression of Gpd1, Fabp4, Ak2, Tpi1 and Ndufa8, scale bar: 200 μm (*n* = 4). (**J**) Oil Red O staining after overexpression of Gpd1, Fabp4, Ak2, Tpi1 and Ndufa8, scale bar: 200 μm (*n* = 4). Values are mean ± SD compared to the control group, * *p* < 0.05, ** *p* < 0.01.

The study found that GPD1, FABP4, AK2, TPI1 and NDUFA8 are closely related to the body’s glucose and lipid metabolism and mitochondrial function. Compared with the control group, after mice were injected with CL316,243, these proteins were also significantly changed in iWAT protein crotonylation modification sequencing (Table S1). Moreover, after CL316,243 treatment of mice, the expression of GPD1 and FABP4 was inhibited, and the expression of AK2, TPI1 and Ndufa8 was promoted (Figure 4A). To further explore the effects of GPD1, FABP4, AK2, TPI1 and NDUFA8 on white fat browning, we constructed their overexpression vectors. The construction and verification of the vectors are shown in Figure S1. 3T3-L1 preadipocytes were induced to differentiate into mature adipocytes, and the constructed overexpression vectors were transferred into mature adipocytes. Cellular RNA was extracted after 24 h, and cellular protein was extracted after 48 h. Quantitative detection found that GPD1 and FABP4 promoted lipid synthesis and inhibited lipolysis and white adipocyte browning (Figure 4B,C); AK2, TPI1 and NDUFA8 inhibited lipid synthesis and promoted lipolysis and white adipocyte browning (Figure 4D–F). The protein level detection results were consistent with the mRNA level detection results (Figure 4G,H). The immunofluorescence detection of UCP1 showed that overexpression of GPD1 and FABP4 inhibited the expression of UCP1, while overexpression of AK2, TPI1 and NDUFA8 promoted the expression of UCP1 (Figure 4I). Further, Oil Red O detection showed that overexpression of GPD1 and FABP4 promoted lipid droplet formation, while overexpression of AK2, TPI1 and NDUFA8 inhibited droplet formation (Figure 4J). Based on the above test results, it is shown that GPD1 and FABP4 promoted lipid synthesis and inhibited the process of lipolysis and white adipocyte browning. AK2, TPI1 and NDUFA8 inhibit lipid synthesis and promote lipolysis and white adipocyte browning.

### 2.5. The Crotonylation Levels of GPD1 and FABP4 Were Downregulated, While the Crotonylation Levels of AK2, TPI1 and NDUFA8 Were Upregulated

**Figure 5:**
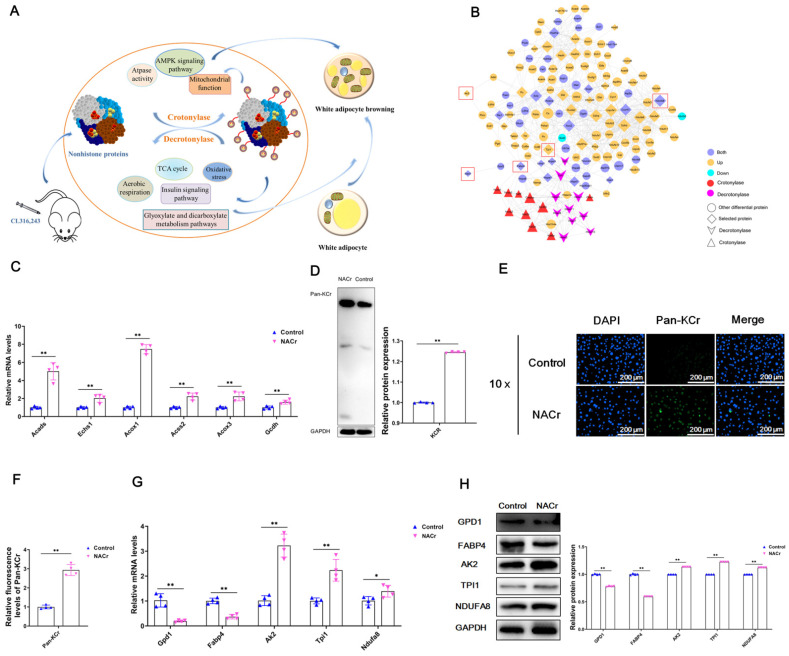
The crotonylation levels of Gpd1 and Fabp4 were downregulated, while the crotonylation levels of Ak2, Tpi1 and Ndufa8 were upregulated. Note: (**A**) Summary figure: after mice were injected with CL316,243, the crotonylation modification of proteins in cells in vivo changed; dynamic regulation of the level of protein crotonylation modification can regulate TCA cycle metabolism, oxidative stress, aerobic respiration, insulin signaling pathway, glyoxylate and dicarboxylic acid metabolism pathway, AMPK signaling pathway, ATPase activity, mitochondrial function, etc., thereby regulating the browning of white fat in the body. (**B**) Interaction network analysis of crotonylated proteins and crotonylation-related enzymes; differentiated and mature adipocytes were treated with 20 mM NACr, and cellular RNA and protein were extracted. (**C**) Relative mRNA expression levels of acylation-related genes after NACr treatment (*n* = 4). (**D**) Expression and quantification of Pan-KCr protein after NACr treatment (*n* = 4). (**E**,**F**): Immunofluorescence and quantitative map of Pan-KCr protein after NACr treatment, scale bar: 200 μm (*n* = 4). (**G**,**H**) The relative mRNA and protein expression levels of Gpd1, Fabp4, Ak2, Tpi1 and Ndufa8 after NACr treatment (*n* = 4). Values are mean ± SD compared to the control group, * *p* < 0.05, ** *p* < 0.01.

Based on the sequencing results, the analysis revealed that after mice were injected with CL316,243, the protein crotonylation modification in vivo was changed. Dynamic regulation of the level of protein crotonylation regulates the metabolic activity of the body, thereby regulating the browning of white fat (Figure 5A).

Protein crotonylation modification omics sequencing of model mouse iWAT revealed that GPD1, FABP4, AK2, TPI1 and NDUFA8 proteins have protein interactions with crotonylase and decrotonylase. The analysis revealed that GPD1 and FABP4 had both up- and downregulated crotonylation sites, while AK2, TPI1 and NDUFA8 were all upregulated crotonylation sites (Figure 5B, Table S1). Therefore, we guessed that GPD1, FABP4, AK2, TPI1 and NDUFA8 were closely related to the level of protein crotonylation.

Next, adipocytes were treated with sodium crotonate (NACr, 20 mM) for 6 h. Quantitative detection showed that crotonylation-related genes were significantly upregulated after NACr treatment (Figure 5C); The results of protein detection showed that lysine crotonylation-modified protein (Pan-KCr) was significantly upregulated after NACr treatment (Figure 5D), which was consistent with the results of mRNA detection. Further immunofluorescence detection of Pan-KCr protein found that after NACr treatment of cells, the fluorescence emphasis of Pan-KCr protein was significantly enhanced (Figure 5E,F). This indicates that NACr treatment can provide a protein crotonylation modification environment for cells. After NACr treatment, further detection showed that the expression levels of GPD1 and FABP4 were downregulated, while the expression levels of AK2, TPI1 and NDUFA8 were all upregulated (Figure 5G,H). The detection results were consistent with the sequencing and previous detection results, indicating that GPD1 and FABP4 proteins were downregulated by crotonylation modification, while AK2, TPI1 and NDUFA8 proteins were all upregulated by crotonylation modification.

### 2.6. Crotonylation Modifications of GPD1, FABP4, AK2, TPI1 and NDUFA8 All Promote White Adipocyte Browning

**Figure 6:**
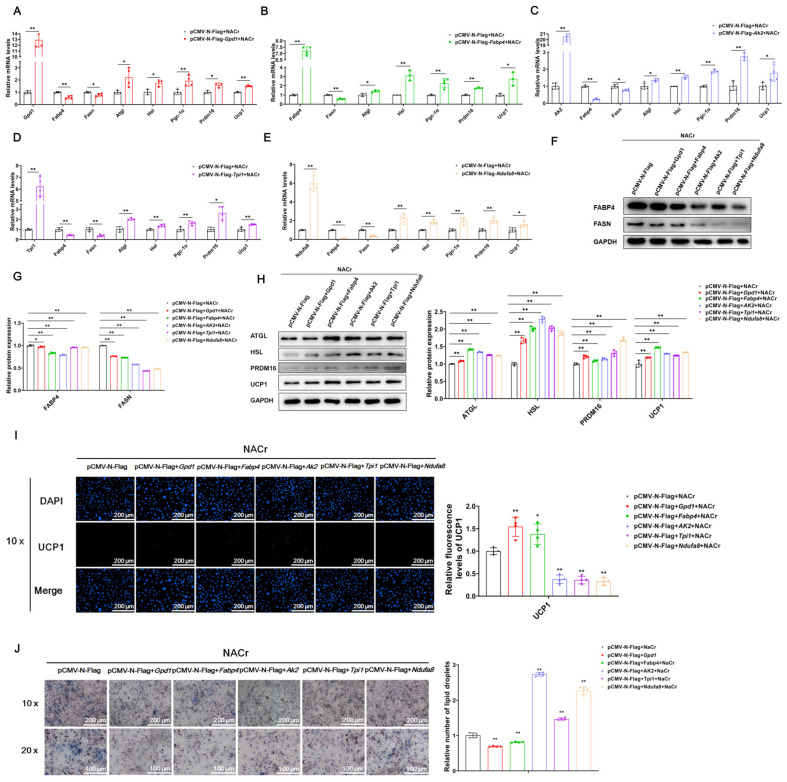
Crotonylation modifications of GPD1, FABP4, AK2, TPI1 and NDUFA8 all promoted white adipocyte browning. Note: 3T3-L1 pre-adipocytes were induced to differentiate into mature adipocytes, and the constructed overexpression vector was transferred into differentiated and mature adipocytes. Cells were treated with NACr, and cellular RNA and protein were extracted 24 h and 48 h after transfection to detect gene overexpression efficiency and relative expression levels of lipid synthesis-related genes Fabp4 and Fasn; lipolysis genes Atgl and Hsl; and white fat browning-related genes Pgc-1α, Prdm16 and Ucp1. (**A**–**E**) After overexpression of Gpd1, Fabp4, Ak2, Tpi1 and Ndufa8 and treatment with NACr, the relative mRNA expression levels of genes related to expression efficiency, lipid synthesis, lipolysis and white fat browning (*n* = 4). (**F**–**H**) Relative protein expression levels of genes related to lipid synthesis, lipolysis and white fat browning after overexpression of Gpd1, Fabp4, Ak2, Tpi1 and Ndufa8 and treatment with NACr (*n* = 3). (**I**) Immunofluorescence staining and quantification of UCP1 after NACr treatment and overexpression of Gpd1, Fabp4, Ak2, Tpi1 and Ndufa8, scale bar: 200 μm (*n* = 4); (**J**) Oil Red O staining after overexpression of Gpd1, Fabp4, Ak2, Tpi1 and Ndufa8 and NACr treatment, scale bar: 200 μm (*n* = 4). Values are mean ± SD compared to the control group, * *p* < 0.05, ** *p* < 0.01.3.

In order to explore the effect of GPD1, FABP4, AK2, TPI1 and NDUFA8 crotonylation on white adipocyte browning, we transfected the overexpression vector into mature adipocytes and treated them with NACr. Cellular RNA was extracted 24 h after transfection, and cellular protein was extracted 48 h after transfection. After NACr treatment, quantitative detection showed that overexpression of GPD1, FABP4, AK2, TPI1 and NDUFA8 inhibited the expression of genes related to lipid synthesis and promoted the expression of genes related to lipolysis and white adipocyte browning (Figure 6A–E); this indicates that GPD1, FABP4, AK2, TPI1 and NDUFA8 crotonylation promotes white adipocyte browning. After NACr treatment, WB detection showed that overexpression of GPD1, FABP4, AK2, TPI1 and NDUFA8 inhibited the expression of lipid synthesis-related proteins and promoted the expression of lipolysis- and white lipid browning-related proteins (Figure 6F–H). The protein level detection results were consistent with the mRNA level detection results. UCP1 immunofluorescence detection showed that after NACr treatment, overexpression of GPD1, FABP4, AK2, TPI1 and NDUFA8 promoted the expression of UCP1 (Figure 6I). Further, Oil Red O detection showed that after NACr treatment, overexpression of GPD1, FABP4, AK2, TPI1 and NDUFA8 inhibited lipid droplet formation (Figure 6J). These results indicated that the crotonylation modifications of GPD1, FABP4, AK2, TPI1 and NDUFA8 inhibited lipid synthesis and promoted lipolysis and white adipocyte browning. 

## 3. Discussion

β3-Adrenergic receptor agonist (CL316,243) can significantly reduce body weight in rodents, promote the decomposition of white adipose tissue, enhance the non-shivering thermogenesis of brown adipose tissue, and reduce blood glucose without affecting food intake. Therefore, it is often used to construct white fat browning model mice. To investigate the effect of protein crotonylation modification on white fat browning, the white fat browning model mice were constructed by intraperitoneal injection of CL316,243 into C57BL/6J.

Crotonyl is structurally similar to acetyl, but there is an extra carbon–carbon double bond, which indicates that the crotonyl group is slightly larger than the acetyl group in spatial structure, and has a stronger transcriptional activation. Its mechanism of action and the acyltransferase and deacyltransferase used are similar to those of acetylation which occurs mainly on the lysine residues of histones [1,34,35]. With the development of proteomics and the application of crotonylation labeling technology, crotonyl modifications are found to exist on non-histone proteins as well [36]. In this study, we focus on the role of non-histone crotonyl modifications in the process of white fat browning in mice. The iWATs of wild-type mice (WT) and white fat browning model mice (CL316,243) are collected, and iWAT non-histone crotonylation in white fat browning model mice is analyzed by proteomic sequencing of crotonylation. KEGG pathway enrichment analysis reveals that crotonylated proteins are significantly enriched in the TCA cycle, glyoxylate and dicarboxylate metabolism, and insulin signaling pathways. This indicates that protein crotonylation is closely related to the metabolic processes in white fat browning mice and is an important protein post-translational modification means to regulate body metabolism, which may be an important entry point for controlling obesity and treating obesity-related metabolic diseases in the future.

Further, we analyze and verify the sequencing results. Firstly, the sequencing results are used to screen the proteins GPD1, FABP4, AK2, TPI1 and NDUFA8, which highly differentially expressed and closely related to the metabolic process. GPD1, FABP4, AK2, TPI1 and NDUFA8 are key enzymes in the energy metabolism process of the body. Meanwhile, in the CL/WT group, the fold change of protein crotonylation modification sequencing difference was large. Therefore, we select these five proteins for subsequent experimental validation. It was found that GPD1 and FABP4 promote lipid synthesis and inhibit lipolysis and white adipocyte browning, while AK2, TPI1 and NDUFA8 inhibit lipid synthesis and promote lipolysis and white adipocyte browning. The roles of GPD1, FABP4, AK2, TPI1 and NDUFA8 in lipid synthesis, lipolysis and white adipocyte browning were preliminarily verified, and the results were also consistent with the results of existing related research.

According to previous studies, NACr is often used as a substrate for crotonylation to catalyze the occurrence of crotonylation modification due to its ability to provide a crotonic acid group [37]. Similarly, we treat differentiated mature adipocytes with 20 mM sodium crotonate for 6 h to construct an environment for intracellular proteins to be modified by crotonylation. It has been found that GPD1 and FABP4 are downregulated, while AK2, TPI1 and NDUFA8 were all upregulated after sodium crotonate treatment. Meanwhile, to investigate the effect of protein crotonylation on the functions of GPD1, FABP4, AK2, TPI1 and NDUFA8, we used sodium crotonate treatment on the basis of overexpression of these genes. The results show that GPD1, FABP4, AK2, TPI1 and NDUFA8 inhibited lipid synthesis and promoted lipolysis and white adipocyte browning after treatment with sodium crotonate. This indicates that the increased level of crotonylation modification inhibited the expression of GPD1 and FABP4, thereby inhibiting their function, and promoted the expression of AK2, TPI1 and NDUFA8, thereby enhancing their function. These findings are consistent with the sequencing results and those of previous studies. The results also showed that changes in the level of crotonylation of GPD1, FABP4, AK2, TPI1 and NDUFA8 could alter their functions to alter their functions in lipid synthesis, lipolysis and browning of white adipocytes. This also means that the function of the target protein can be regulated by controlling the crotonylation level of the target protein, which has far-reaching significance for the study of obesity control and related diseases and provides a theoretical basis and experimental basis for the production of related drugs.

## 4. Materials and Methods

### 4.1. Animal Experiment

Eight-week-old C57BL/6J male mice were purchased from the Experimental Animal Center of Air Force Military Medical University (Xi’an, China). Mice rearing and handling were carried out in accordance with the guidelines and regulations approved by the Animal Ethics Committee of Northwest Agriculture and Forestry University (Yangling, Shaanxi). Mice were housed at 5 per cage, drinking ad libitum, and kept on standard laboratory chow. The animal room was maintained at a constant temperature of 25 ± 1 °C, a humidity of 55 ± 5%, and a 12 h light/12 h dark cycle.

After two weeks of feeding, C57BL/6J male mice were injected intraperitoneally with CL316,243 (1 mg/kg, Sigma-Aldrich, Shanghai, China) every two days for 4 weeks. The mice were stabilized for another 2 weeks, and inguinal white adipose tissue (iWAT) was collected from the mice for subsequent experiments.

### 4.2. LC-MS/MS Analysis

LC-MS/MS analysis was performed by the commissioned Jingjie Biological Technology Co., Ltd. (Hangzhou, China). The processes involved are protein extraction, pancreatase enzymes, affinity enrichment, liquid chromatography–mass spectrometry series analysis, database search and biological information analysis.

### 4.3. Real-Time Quantitative PCR (qPCR)

Total RNA was extracted from mouse inguinal subcutaneous white adipose tissue (iWAT) or adipocytes, and 500 ng of total RNA was reverse transcribed using M-MLV reverse transcriptase kit (Takara, Dalian, China). All primers were synthesized by Qingke Biotechnology (Qingke, Beijing, China). qPCR was performed in a 10 μL reaction system containing the specific primers and AceQ qPCR SYBR Green Master Mix (Vazyme, Nanjing, China). Each sample had 4 replicates, with actin as the internal reference, and the quantitative data were analyzed using 2^−ΔΔCt^, ΔΔ = (CT _gene_ − CT _actin_) T − (CT _gen e_− CT _actin_) C. The involved qPCR primers and amplified cDNA primers are shown in Tables S1 and S2.

### 4.4. Western Blotting (WB)

Total protein (30 μg) was extracted from iWAT or adipocytes, separated by SDS-PAGE and transferred to PVDF nitrocellulose membrane (Millipore, Boston, MA, USA). After being blocked with 5% nonfat milk for 2 h at room temperature, the primary antibody was incubated for 2 h at room temperature. The antibodies used in the WB process are: Pan-KCr (PTM BIO, Hangzhou, China), FLAG (Abways, Shanghai, China), UCP1 (Beyotime, Shanghai, China), PRDM16 (Beyotime, Shanghai, China), ATLGL (Beyotime, Shanghai, China), FASN (Abways, Shanghai, China), FABP4 (Beyotime, Shanghai, China), GPD1 (Abcam, Shanghai, China), NDUFA8 (Abways, Shanghai, China), TPI1 (Abways, Shanghai, China), AK2 (Abways, Shanghai, China), GAPDH (Abways, Shanghai, China). Mouse or rabbit HRP-conjugated secondary antibodies (Baoshen, Beijing, China) were added and incubated for 1 h at room temperature. Proteins were visualized using chemiluminescent peroxidase substrate (Millipore, Boston, MA, USA) and the ChemiDoc XRS system (Bio-Rad, Hercules, CA, USA), and the blots were quantitatively analyzed using ImageJ (ij142-jdk6-setup) software.

### 4.5. Immunofluorescence

Cells were first treated for 48 h as required for the experiment, fixed with 4% paraformaldehyde (MACKLIN, Shanghai, China), permeabilized with 0.1% Triton x-100 (Solarbio, Beijing, China) and then blocked with 5% bovine serum albumin (BSA, WOLSEN, Shenzhen, China) for 1 h at room temperature. The cells were incubated with UCP1 (Beyotime, Shanghai, China) or FLAG (Abways, Shanghai, China) for 1 h at room temperature, followed by incubation with goat anti-rabbit IgG antibody (Boster, Wuhan, China) coupled with fluorescein isothiocyanate for 1 h at room temperature, and stained with DAPI (Solarbio, Beijing, China) for 5 min. Cells were observed and photographed using the Cytation3 Cell Imaging Multi-Mode Reader (BioTek, Winooski, VT, USA).

### 4.6. HE Staining of Tissue Sections

The paraffin sections were successively immersed in xylene solution; absolute ethanol; and 90%, 80%, and 70% alcohol for 5 min each for dewaxing and rehydration. They were then stained with hematoxylin for 5 min, differentiated with 5% acetic acid for 1 min and returned to blue with a blue-returning solution. After 1 min of eosin staining, gradient dehydration was carried out by immersion in 70%, 80% and 90% alcohol and absolute ethanol for 10 s and soaking in xylene for 1 min. Finally, after drying and mounting, microscopic examinations were carried out.

### 4.7. Oil Red O Staining

Mature adipocytes differentiated from 3T3-L1 were stained using an Oil Red O staining kit (Solebold, Beijing, China). The cells were first fixed with ORO Fixative according to the instructions and then stained by adding a newly prepared ORO Stain staining solution. Mayer hematoxylin solution was added to stain the cell nuclei, and the cells were finally observed under the microscope.

### 4.8. Culture and Induced Differentiation of 3T3-L1 Adipose Precursor Cells

The cryopreserved 3T3-L1 adipocytes were recovered, passaged and planked. Taking the 6-well plate as an example, when the cell density reached about 90%, after contact inhibition for 2 days, inducible solution I (IBMX: 0.5 mmol/L, TOPSCIENCE, Shanghai, China; Dex: 1 μmol/L, Solarbio, Beijing, China; insulin: 10 μg/mL, TOPSCIENCE, Shanghai, China) was added to the culture for 2 days, then inducible solution II (insulin: 10ug/mL) was added to the culture for 8–10 days, and inducible solution II was changed every 2 days. After that, the medium was replaced with 10% serum double antibody-free medium, and the cells were transfected after 24 h of culture. We added 2 μg of recombinant plasmid and 2 μL of lipo2000 (Invitrogen, Waltham, MA, USA) to the Opti (Thermo Fisher, Shanghai, China), mixed them by vortexing, and let the solution stand for 20 min. We added the mixed solution to a Petri dish, put it in the incubator for 6 h and then replaced it with normal culture medium. After 24 or 48 h, cells were collected for further processing.

### 4.9. Statistical Analysis

All data were analyzed for significant differences using one-way ANOVA and two-way ANOVA. Individual means were compared using Fisher’s least significant difference (LSD). Data are presented as mean ± standard deviation, and *p* < 0.05 was considered significant.

## Supplementary Materials

The supporting information can be downloaded at: https://www.mdpi.com/article/10.3390/ijms232112733/s1.
