# Supplementary material for: Non-Histone Lysine Crotonylation Is Involved in the Regulation of White Fat Browning
Source: Int J Mol Sci. 2022 Oct 22;23(21):12733. doi: 10.3390/ijms232112733 (PMC9658748; doi:10.3390/ijms232112733)
Supplement: Supplementary file 1 [file ijms-23-12733-s001.zip › ijms-1921775-supplementary.pdf]

Appendix: additional image and table

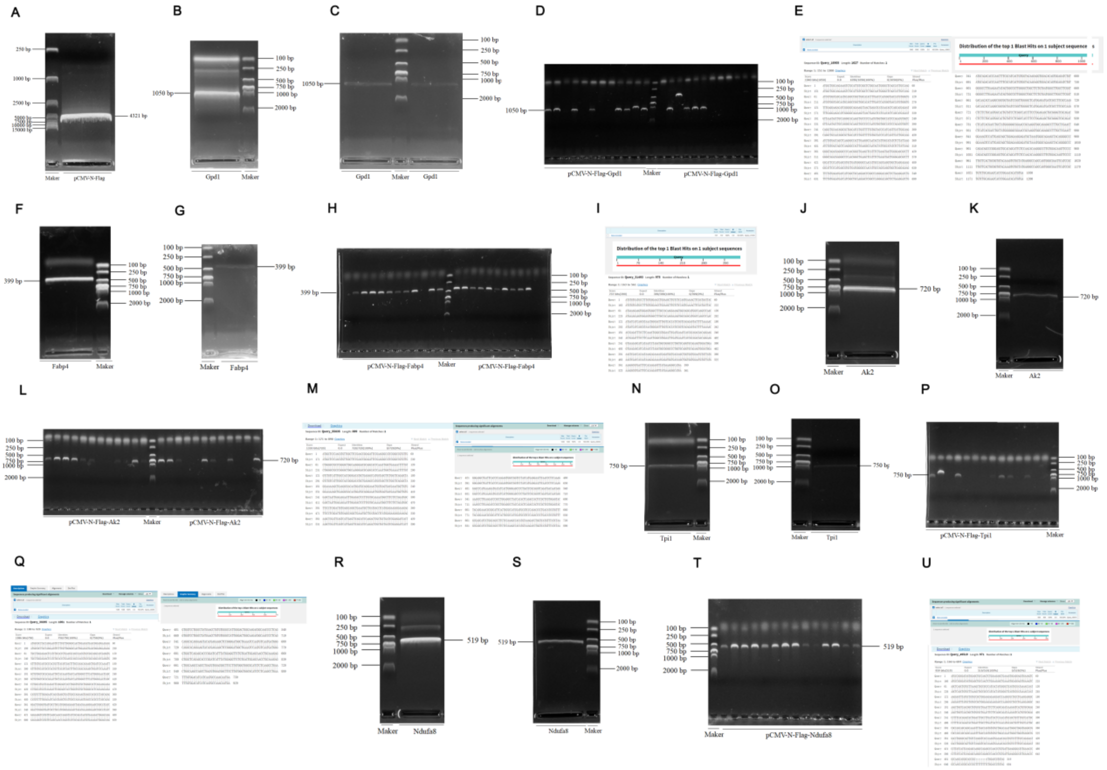

Figure S1 Construction of overexpression vector for related target genes

Note: A: The pCMV-N-Flag overexpression vector was recovered by double-enzyme digestion and purified gel; B-E: Construction and sequencing verification of pCMV-N-Flag-Gpd1 overexpression vector; F-I: Construction and sequencing verification of pCMV-N-Flag-Fabp4 overexpression vector; J-M: Constructio quencing verification of pCMV-N-Flag-Ak2 overexpression vector; N-Q: Construction and sequencing verification of pCMV-N-Flag-Tpi1 overexpression vector; R-U: Construction and sequencing verification of pCMV-N-Flag-Ndufa8 overexpression vector.

Table S1 Identification of normalized modification Kcr sites quantitation on non-histone proteins in CL mice

Protein accession	Protein description	Gene name	Regulated Type	Amino acid	Kcr sites	Normalized modification sites quantitation
						Kcr Ratio(CL/WT)
P13707	Glycerol-3-phosphate dehydrogenase [NAD(+)], cytoplasmic	GPD1	Down	K	130	0.5626703
P13707	Glycerol-3-phosphate dehydrogenase [NAD(+)], cytoplasmic	GPD1	Down	K	313	0.828175896
P13707	Glycerol-3-phosphate dehydrogenase [NAD(+)], cytoplasmic	GPD1	Down	K	178	0.639287766
P04117	Fatty acid-binding protein, adipocyte	FABP4	Down	K	97	0.570131639

Q9WTP 6	Adenylate kinase 2, mitochondrial	AK2	Up	K	160	1.345023697
P17751	Triosephosphate isomerase	TPI1	Up	K	238	2.08
P17751	Triosephosphate isomerase	TPI1	Up	K	210	1.528880866
P17751	Triosephosphate isomerase	TPI1	Up	K	199	2.01.396915584
P17751	Triosephosphate isomerase	TPI1	Up	K	56	1.50371471
Q9DCJ5	NADH dehydrogenase [ubiquinone] 1 alpha subcomplex subunit 8	NDUFA8	Up	K	159	1.749373434
Q9DCJ5	NADH dehydrogenase [ubiquinone] 1 alpha subcomplex subunit 8	NDUFA8	Up	K	162	1.922965116
Q9DCJ5	NADH dehydrogenase [ubiquinone] 1 alpha subcomplex subunit 8	NDUFA8	Up	K	132	1.441558442
Q9DCJ5	NADH dehydrogenase [ubiquinone] 1 alpha subcomplex subunit 8	NDUFA8	Up	K	41	1.593824228
